# Characterization and quantification of tumor infiltrating lymphocytes in breast cancer

**DOI:** 10.1186/2051-1426-2-S1-P9

**Published:** 2014-02-24

**Authors:** Shahinaz Bedri, Mahmoud Mohamed, Hina Sarwath, Konduru Sastry

**Affiliations:** 1 Department of Research, Weill Cornell Medical College in Qatar, Doha, Qatar; 2 Pharmacy, Hamad Medical Corporation, Doha, Qatar

## Background

Tumor infiltrating lymphocytes (TILs) are a significant component of the tumor microenvironment [1]. In breast cancer the prognostic and predictive value of TIL is under active investigation [2]. The goal of our study was to define specific TIL subtypes and regional quantification in breast cancer of patients from the MENA region.

## Methodology

47 FFPE samples with known clinic-pathological data were selected and studied using immunohistochemistry technique. TIL immune markers studied were CD3, CD8, CD45RO and FOXP3 and quantified by modified H-score system. Results were analyzed via SPSS.

## Results

A positive correlation between CD3 and CD8 expression (rs=0.614, n=24, P 0.001) and CD3 and CD45RO expression (rs=0.621, n=24, P 0.001) were noted in the center of the tumor (CT).CD3 median percentage in CT was higher in grade 2 and grade 3 breast carcinoma compared to grade 1 (P 0.023). CD3 in the center of the tumor and CD8 in the invasive margins (IM) were significantly higher in ER/PR negative Her2/neu positive tumors compared to triple negative breast cancers. Lower CD3, CD8 and FOXP3 percentages were noticed in TNBC tumors. CD45RO median percentage was shown to be higher in the IM region.

## Conclusion

TIL were affected by the patient clinic-pathological characteristics.
